# Immunohistochemistry Evaluation of the Effect in Vivo of Tumor Necrosis Factor (TNF)-α on Blood Vessel Density in Murine Fibrosarcoma

**DOI:** 10.1080/13577140310001607275

**Published:** 2003-06

**Authors:** Avi Eisenthal, Ignat Schwartz, Josephine Issakov, Yossef Klausner, Faina Misonzhnik, Beatriz Lifschitz-Mercer

**Affiliations:** 1 Pathology Institute Tel-Aviv Sourasky Medical Center The Sackler Faculty of Medicine Tel-Aviv University Tel-Aviv 64239 Israel; 2 Department of Surgery B Tel-Aviv Sourasky Medical Center The Sackler Faculty of Medicine Tel-Aviv University Tel-Aviv Israel

## Abstract

*Purpose*: Angiogenesis is essential for tumor growth and metastases, thus bestowing obvious importance upon methodologies which could enable its inhibition.

*Materials*: C57BL/6 female mice bearing a subcutaneous (s.c.) MCA205 fibrosarcoma were used.

*Methods*: Ten mice were divided equally into two groups. One group was injected intraperitoneally (i.p.) with 10 μg tumor necrosis factor-α (TNF-α and the other (controls) with Hanks balanced salt solution (HBSS). Tumor growth was monitored at least twice weekly. The number of endothelial cells in the blood microvessels was assessed by immunohistostaining on paraffin-embedded tumor tissue sections using vascular endothelial growth factor (VEGF) and Factor 8 antibodies. Expression of the p53 gene was similarly assessed by immunohistostaining.

*Results*: Injection of 10 μg TNF-α into the tumor-bearing mice reduced the number of endothelial cells in the blood microvessels by 46% on day 3 post-injection which was accompanied by an increase (by 37%) in the expression of p53 in these cells. It also inhibited tumor growth compared to the HBSS-injected group starting at 17 days post-cytokine injection.

*Discussion*: The antitumor *in vivo* effect exerted by TNF-α on established murine sarcoma s.c. tumors may be due to an earlier effect of the cytokine on the tumor's blood microvessels, probably through an apoptotic mechanism involving the p53 gene.


*Sarcoma*, June 2003, *VOL. 7, NO. 2, 57–61*

Taylor & Francis healthsciences

ORIGINAL ARTICLE

### Immunohistochemistry evaluation of the effect in vivo of tumor necrosis factor (TNF)-α on blood vessel density in murine fibrosarcoma

AVI EISENTHAL[1], IGNAT SCHWARTZ[1], JOSEPHINE ISSAKOV[1], YOSSEF KLAUSNER[2], FAINA MISONZHNIK[1] & BEATRIZ LIFSCHITZ-MERCER[1]

[1]*Pathology Institute &* [2]*Department of Surgery B, Tel-Aviv Sourasky Medical Center, affiliated to the Sackler Faculty of Medicine, Tel-Aviv University, Tel-Aviv, Israel*

**Abstract**

*Purpose*: Angiogenesis is essential for tumor growth and metastases, thus bestowing obvious importance upon methodologies which could enable its inhibition.
*Materials*: C57BL/6 female mice bearing a subcutaneous (s.c.) MCA205 fibrosarcoma were used.
*Methods*: Ten mice were divided equally into two groups. One group was injected intraperitoneally (i.p.) with 10 μg tumor necrosis factor-α (TNF-α and the other (controls) with Hanks balanced salt solution (HBSS). Tumor growth was monitored at least twice weekly. The number of endothelial cells in the blood microvessels was assessed by immunohistostaining on paraffin-embedded tumor tissue sections using vascular endothelial growth factor (VEGF) and Factor 8 antibodies. Expression of the p53 gene was similarly assessed by immunohistostaining.
*Results*: Injection of 10 μg TNF-α into the tumor-bearing mice reduced the number of endothelial cells in the blood microvessels by 46% on day 3 post-injection which was accompanied by an increase (by 37%) in the expression of p53 in these cells. It also inhibited tumor growth compared to the HBSS-injected group starting at 17 days post-cytokine injection.
*Discussion*: The antitumor *in vivo* effect exerted by TNF-α on established murine sarcoma s.c. tumors may be due to an earlier effect of the cytokine on the tumor's blood microvessels, probably through an apoptotic mechanism involving the p53 gene.

### Introduction

Angiogenesis plays an important role in the growth, progression and metastases of different solid tumors, including melanoma and carcinoma of breast, lung, colon and prostate.[1,2] Moreover, the number of blood microvessels in tumor tissues has been shown to be of prognostic value for certain tumors.[2] Thus, the inhibition of angiogenesis is considered to be highly effective for assessing the growth of solid tumors.

Tumor angiogenesis is controlled by a number of positive and negative regulators that are elaborated by tumor cells and tumor-associated host cells, particularly by tumor-associated macrophages. Such regulators include basic fibroblast growth factor (bFGF), vascular endothelial growth factor (VEGF), transforming growth factor (TGF)-$\beta$, platelet-derived growth factor (PDGF), interleukin (IL)-8 and thrombospondin-1.[3,4]

Another molecule which regulates angiogenesis is tumor necrosis factor (TNF)-α. This molecule was originally described as a protein that mediates a primary hemorrhagic necrosis and the subsequent regression of established subcutaneous (s.c.) tumors.[5] Such antitumor effects, which usually occur within a short period following injection of TNF-α, imply that TNF-α may be involved in angiogenesis by destroying the tumor's blood microvessels, thus leading to hypoxia followed by activation of the p53 gene and ultimately to apoptosis.[6] The evidence in support of such a possibility, however, is controversial. In some reported studies, the secretion of TNF-α by activated monocytes induced angiogenesis via the production of IL-8, VEGF and bFGF4, while other studies showed either no effect of TNF-α on microvessel density[7] or an anti-angiogenic effect when TNF-α was combined with linomide[8] and lovastatin.[9]

Similar to what was shown to occur in carcinoma and melanoma, modulation of angiogenesis in mesenchymal tumors, such as fibrosarcoma 105 and HT1080, has been demonstrated using the AGM-1470 and thrombospondin-1 modulators,

Correspondence to: Avi Eisenthal, PhD, Director, Pathology Laboratory, Tel-Aviv Sourasky Medical Center, 6 Weizman Street, Tel-Aviv, 64239 Israel. Tel: +972-3-697-4645; Fax: +972-3-697-4648; E-mail: eisenthal@tasmc.health.gov.il

ISSN 1357-714X print/ISSN 1369-1643 © 2003 Taylor & Francis Ltd
DOI: 10.1080/13577140310001607275

respectively.[3,10] The effect of TNF-α on mesenchymal tumors, however, remains unknown.

In the present study, we used an immunohistochemistry method to analyze the effect *in vivo* of TNF-α on the number of endothelial cells in the blood microvessels of tumors in a murine fibrosarcoma model. Our results demonstrate that an injection of TNF-α at the same dose which had reduced the growth rate of established s.c. MCA205 tumors in mice[11] substantially diminished the number of endothelial cells in the blood microvessels in that tumor.

### Materials and methods

### *Animals*

Female C57BL/6 mice were obtained from the Animal Facility Building at the Tel-Aviv University and were used when they were 12–20 weeks old.

### *Tumor*

A MCA205 fibrosarcoma line, syngeneic to C57BL/6 ($H2^b$) mice, was kindly provided by the Surgery Branch, National Cancer Institute, Bethesda, MD, USA. These cells were grown in culture and underwent passages twice weekly. Before use, the MCA205 cells were harvested by exposure for 2–3 min to trypsin–EDTA followed by cell washing in complete medium (CM). The CM consisted of RPMI 1640 with 10% heat-inactivated fetal calf serum (FCS), 0.1 mM nonessential amino acids, 1 mM sodium pyruvate, 100 μg streptomycin, 100 U/ml penicillin, 0.03% fresh glutamine, 50 μg/ml gentamicin and 0.5 μg/ml Fungizone. All the products were purchased from Biological Industries, Israel.

### *Cytokine*

Recombinant TNF-α was purchased from PeproTech, NJ, USA (catalog #300-01A). This material has a specific activity of $>2 \times 10^7$ units/mg protein determined by the cytolysis of murine L929 cells in the presence of actinomycin D, and endotoxin levels below 0.1 ng/μg of rTNF-α.

### In vivo *model*

MCA205 tumor cells were injected s.c. into the animal's abdomen with $5 \times 10^5$ cells/0.1 ml Hanks balanced salt solution (HBSS). When the tumors were palpable, the mice were divided into two groups: those in the first group were injected intraperitoneally (i.p.) with TNF-α at 10 μg/0.5 ml HBSS and those in the second group were injected i.p. with HBSS alone. Tumor growth was determined in a blinded fashion throughout the entire study period by the same investigator who recorded the largest perpendicular diameters (the multiplication of which is referred to as 'tumor area').[11] Tumor growth was monitored at least twice weekly.

### *Immunohistostaining*[12]

Tumor tissues were fixed in formalin and subjected to increasing concentrations of ethanol and xylene. The tissue was embedded in paraffin, cut into sections 3–5 μm thick and incubated with the following antibodies: VEGF (Santa Cruz, CA, USA, catalog #SC7269, dilution 1: 200), F8 (Zymed, CA, USA catalog #18-0018, dilution 1: 50) and p53 (Zymed, catalog #18-0129, dilution 1: 40). The tissue sections were then incubated with biotin-labeled goat anti-mouse antibody (Ventana, AZ, USA) followed by exposure to avidin–peroxidase complex (Ventana). Finally, diaminobenzidine (DAB, Ventana) was added to serve as a substrate.

### *Screening of stained tissues for positive cells*[12]

Each stained tissue was screened in a blinded fashion under a microscope at $\times 400$ magnification ($0.4\ mm^2$/field). For each tissue section, 25 different fields were randomly selected and the number of blood microvessels, assessed by identifying stained endothelial cells, was scored by the same investigator throughout the entire study. To avoid false-positive staining, fields were selected at a relative distance from tissue section margins.

### Results

### *Effect of TNF on MCA205 tumor growth*

Ten mice were injected s.c. with $5 \times 10^5$ MCA205 tumor cells. When tumors were palpable (usually on days 7–10 after injection of the cells), the mice were equally divided into two groups. The mice in one group were injected i.p. with 10 μg/0.5 ml TNF, a dose which had been found in a previous study to exert an antitumor activity on established s.c. tumors in mice.[13] The mice in the second group which served as controls were injected with 0.5 ml HBSS. As shown in Fig. 1, no difference was noted in tumor growth rate between the TNF and HBSS groups up to day 19 after TNF injection. Thereafter, the growth rate of tumor in the HBSS control group was substantially higher than that in the TNF group. Based on these findings, an i.p. injection of TNF at 10 μg/mouse was selected for use throughout the entire study.

### *Modulation of the number of blood microvessel endothelial cells in the MCA205 tumors by TNF-α*

An immunohistochemistry method using VEGF and F8 antibodies which react with endothelial cells was

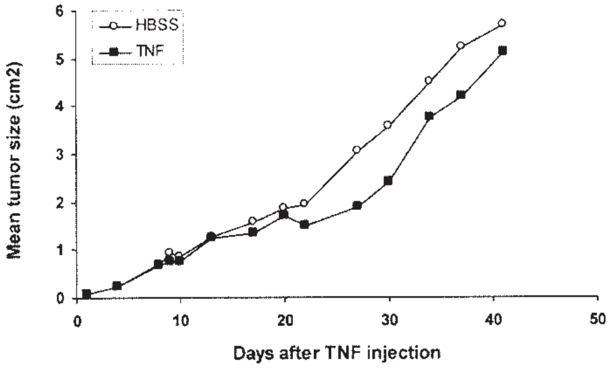

**Fig. 1.** *Effect of TNF-α on the growth of MCA205 tumor. Ten C57BL/6 mice were injected s.c. with 5 × 10⁵ MCA205 tumor cells. When the tumors were palpable, the mice were injected i.p with either 0.5 ml HBSS or 10 μg TNF-α/0.5 ml HBSS. Each dot represents the mean of five mice at each time point.*

employed to evaluate the effect of TNF-α on the number of blood microvessels in the MCA205 tumors. The mice bearing palpable tumors (usually 7–10 days post tumor cells injection) were injected i.p. with 10 μg/ml TNF. The control mice were injected in the same manner with HBSS. The animals were sacrificed at 1, 3 and 7 days after TNF-α injection, and the tumor was removed and fixed in formalin. After they were embedded, the tumor tissues were cut and immunohistostained with VEGF and F8 antibody which specifically reacts with the endothelial cells in the blood microvessels. The mean number (three mice in each group) ± standard error of the mean (SEM) of blood microvessels, assessed by F8-positive endothelial cells in the HBSS-injected mice was 29.3 ± 7.2, 63.3 ± 15.6 and 73.3 ± 10.0 at 1, 3 and 7 days post-injection, respectively (Fig. 2). In the TNF-α-injected mice, the mean number of blood microvessels assessed by F8-positive endothelial cells was 31.7 ± 10.0, 34.3 ± 2.5 ($P < 0.05$ compared to HBSS group) and 51.0 ± 8.6 ($P < 0.05$ compared to HBSS group) at 1, 3 and 7 days post-injection, respectively. Similarly, when VEGF was used for identifying endothelial cells, the mean number of blood microvessels in the HBSS-injected mice increased from 28.0 ± 6.1 at day 1 to 50.7 ± 10.2 and 57.3 ± 0.6 at 3 and 7 days post-injection, respectively (Fig. 3), while in the TNF-α-injected mice, the number of blood microvessels increased from 27.3 ± 10.2 at day 1 to 27.3 ± 8.4 ($P < 0.05$ compared to HBSS group) and 44.7 ± 0.6 at 3 and 7 days post-injection, respectively.

*Effect of TNF-α on the expression of p53 in MCA205 tumors*

It has been shown that inhibition of angiogenesis causes apoptosis of endothelial cells via the activation of the p53 suppressor gene.[6] Since TNF-α was found in previous experiments to reduce the number of

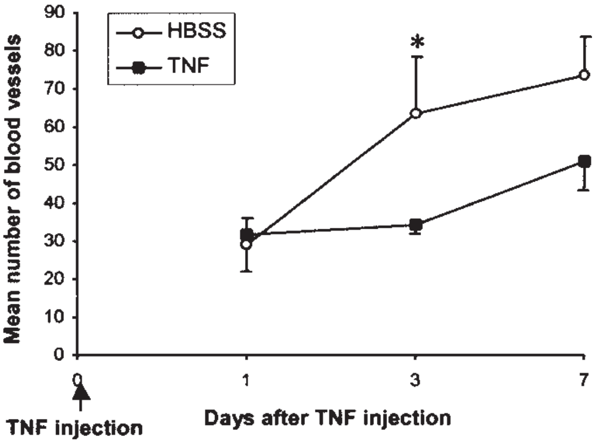

**Fig. 2.** *Effect of TNF-α on the number of blood microvessels assessed by Factor 8 antibody staining. C57BL/6 mice were injected s.c. with 5 × 10⁵ MCA205 tumor cells followed by an i.p. single injection of either 10 μg TNF-α or HBSS. On days 1, 3 or 7, the mice were sacrificed, the tumors removed, and the paraffin-embedded tissue sections were stained with F8 antibody. Each dot represents the mean number (including the standard error of the mean [SEM]) of the blood microvessels calculated from tumors resected from three different mice *P < 0.05.*

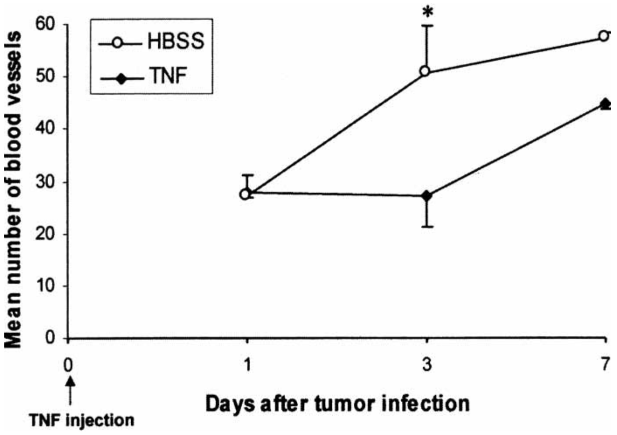

**Fig. 3.** *Effect of TNF-α on the number of blood microvessels assessed by VEGF antibody staining. C57BL/6 mice were injected s.c. with 5 × 10⁵ MCA205 tumor cells followed by an i.p. single injection of either 10 μg TNF-α or HBSS. On days 1, 3 or 7, the mice were sacrificed, the tumors removed and the paraffin-embedded tissue sections were stained with VEGF antibody. Each dot represents the mean number (including the standard error of the mean [SEM]) of the blood microvessels calculated from tumors resected from three different mice *P < 0.05.*

endothelial cells in the blood microvessels in MCA205 tumors, the possible involvement of p53 in this phenomenon was also addressed. For that purpose, mice were injected with either TNF-α or HBSS, sacrificed at various time intervals, the tumor was removed, embedded in paraffin and then immunohistostained with p53 antibodies. The intensity of staining in endothelial cells of blood microvessels was determined as following: +1 = low intensity, +2 = medium intensity and +3 = high intensity. As shown in Fig. 4, TNF-α induced a greater increase than HBSS in the expression of p53

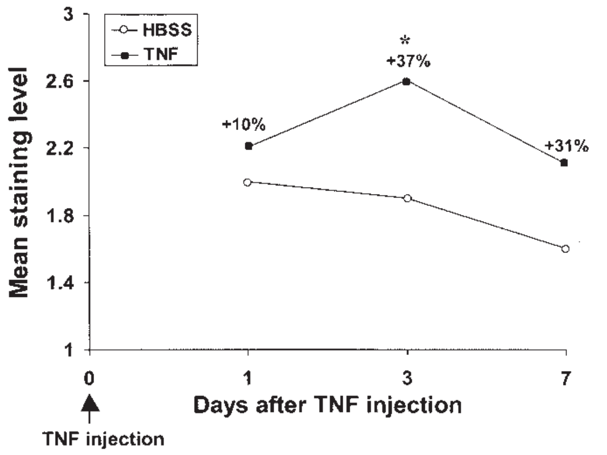

**Fig. 4.** *Effect of TNF-α on the expression of p53 protein in the blood microvessels endothelial cells. C57BL/6 mice were injected s.c. with 5 × 10⁵ MCA205 tumor cells followed by an i.p. single injection of either 10 μg TNF-α or HBSS. On day 1, 3 or 7, the mice were sacrificed, the tumors removed, and paraffin-embedded tissue sections were stained with p53 antibody. Each dot represents the mean expression of p53, presented as the staining level, calculated from tumors resected from three different mice. *P < 0.05. The indicated numbers show the percent increase in p53 expression over control mice injected with HBSS.*

protein in blood microvessel endothelial cells. At day 1 the increase in the mean staining level in TNF-α compared to HBSS-injected mice was from 2.0 to 2.2 at day 1 (10% increase), from 1.9 to 2.6 (37% increase) at day 3 and from 1.6 to 2.1 (31%) at day 7.

### Discussion

Malignant soft tissue tumors, sarcomas, account for less than 1% of all invasive neoplasms, but they often exhibit highly malignant behavior and cause at least 20% of all deaths from malignant diseases.[21] Among the different factors which modulate the growth of tumors, the generation of new blood vessels in the tumor, a process known as angiogenesis, plays a pivotal role.[1,2] Thus, the ability to modulate this process is enormously important.

In the present study, we tested the ability of TNF-α to modulate the endothelial cells in the blood microvessels of the MCA205 fibrosarcoma murine tumor. Our results showed that an i.p. injection of 10 μg TNF-α caused a substantial reduction in the number of tumor blood microvessels compared to the number of blood microvessels counted in the tumor-bearing mice which received HBSS alone. A maximal reduction of 46% was observed 3 days post-TNF-α injection, as assessed by both VEGF and Factor 8 antibodies. Furthermore, monitoring tumor growth following TNF-α injection revealed a substantial reduction in tumor growth rate, starting 19 days post-cytokine injection, compared to the HBSS-treated control mice. Others have shown that TNF may cause the necrosis of s.c. tumors within hours following the injection of the cytokine.[14] Such a rapid response may be the result of employing higher concentrations of the cytokine or a different route of injection, e.g., intravenously. It is also possible that the effect of TNF in our study was due to a delayed antitumor cell-mediated immune response.[15] In order to further explore this possibility, experiments aimed at delineating the role of a cell-mediated immune response in the described experimental model are currently being conducted on mice with an impaired immune system.

In contrast to our current findings, other investigators have failed to demonstrate any effect of TNF-α on tumor vascularization or to show an increase in angiogenesis.[4,7–9] Such differences in results could be explained by the dose, injection regimen and route of administration of the cytokine, all of which may affect the concentration of the cytokine reaching the tumor site. It is also possible that endothelial cells in various tumors may differ in the expression of specific receptors for TNF-α,[16] a feature which would affect their sensitivity to TNF-α activity. Alternatively, since tumor-associated macrophages (TAM) were shown to modulate angiogenesis via the secretion of IL-8, VEGF, GM-CSF, IL-1α and TNF-α,[4,8,17] changes in the number of these cells within various tumors or their activation levels may modulate the effect of TNF-α on tumor angiogenesis following its injection into tumor-bearing mice.

The mechanisms involved in the herein described effects of TNF-α on the endothelial cells in the blood microvessels is not fully understood. One possible mechanism is the destruction of endothelial cells through apoptosis: this is supported by our findings demonstrating that TNF-α increased the expression of p53 in the endothelial cells in the blood microvessels by up to 37% when compared to HBSS-injected mice at 3 days post-injections. These findings, however, do not exclude the possibility that TNF-α exerts a cytotoxic signals via a different mechanism, including the generation of free oxygen radicals[18] which were shown to affect the endothelial cells in the blood microvessels[19] by activation of the nuclear factor-κ B.[20]

We demonstrated the effect of TNF-α on an established murine fibrosarcoma line and contend that this was probably through the modulation of the growth of the endothelial cells in the blood microvessels. Experiments to delineate the combined effect of TNF-α with other modulators as well as the mechanism by which this cytokine regulates blood microvessels endothelial cells in murine sarcoma are currently underway.

### Acknowledgment

Esther Eshkol is thanked for editorial assistance.

### References

1. Hanahan D, Folkman J. Pattern and emerging mechanisms of angiogenic switch during tumorigenesis. *Cell* 1996; 86: 353–64.

2. Pluda JM. Tumor-associated angiogenesis: mechanisms, clinical implications and therapeutic strategies. *Semin Oncol* 1997; 24: 203–18.
3. Volpert OV, Lawler J, Bouck NP. A human fibrosarcoma inhibits systemic angiogenesis and the growth of experimental metastases via thrombospondin-1. *Proc Natl Acad Sci USA* 1998; 95: 6343–8.
4. Torishu H, Ono M, Kiryu H, Furue M, Ohmoto Y, Nakayama J, Nishioka Y, Sone S, Kuwano M. Macrophage infiltration correlates with tumor stage and angiogenesis in human malignant melanoma: possible involvement of TNF alpha and IL-1 alpha. *Int J Cancer* 2000; 85: 182–8.
5. Carswell EA, Old LJ, Kassel RC, Green S, Fiore N, Wiliamson BD. An endotoxin-induced serum factor that causes necrosis of tumors. *Proc Natl Acad Sci USA* 1975; 72: 3666–70.
6. Soengas MS, Alarcon RM, Yoshida H, Giaccia AJ, Hakem R, Mak TW, Lowe SW. Apaf-1 and caspase-9 in p53 dependent apoptosis and tumor inhibition. *Science* 1999; 284: 156–9.
7. Yoshimura H, Chikamoto A, Honda T, Tashiro K, Nakamoto T, Takano M, Takagi K, Nagasue N, Soma G. Relation between microvessel quantification and inducibility of endogenous tumor necrosis factor in colorectal adenocarcinoma. *Anticancer Res* 2000; 20: 629–33.
8. Joseph IB, Isaacs JT. Macrophages role in anti-prostate cancer response to one class of antiangiogenic agents. *J Natl Cancer Inst* 1998; 90: 1648–53.
9. Feleszko W, Balkowiec EZ, Sieberth E, Marczak M, Dabrowska A, Giermasz A, Czajka A, Jakobisiak M. Lovastatin and tumor necrosis factor-alpha exhibit potentiated antitumor effects against Ha-ras-transformed murine tumor via inhibition of tumor-induced angiogenesis. *Int J Cancer* 1999; 81: 560–7.
10. Brem H, Folkman J. Analysis of experimental anti-angiogenic therapy. *J Pediatr Surg* 1993; 28: 445–50.
11. McIntosh JK, Mule JJ, Merino MJ, Rosenberg SA. Synergistic antitumor effects of immunotherapy with recombinant interleukin-2 and recombinant tumor necrosis factor-α. *Cancer Res* 1988; 48: 4011–17.
12. Eisenthal A, Polyvkin N, Bramante-Schreiber L, Misonznik F, Hasner A, Lifschitz-Mercer B. Expression of dendritic cells (DC) in ovarian tumors correlates with the clinical outcome in ovarian cancer patients. *Hum Pathol* 2001; 32: 803–7.
13. Gutman M, Sofer D, Lev-Chelouche D, Merimsky O, Klausner JM. Synergism of tumor necrosis factor-α and melphalan in systemic and regional administration: animal study. *Invasion Metastasis* 1997; 17: 169–75.
14. Asher AL, Mule JJ, Reichert CM, Shiloni E, Rosenberg SA. Studies of the anti-tumor efficacy of systemically administered recombinant tumor necrosis factor against several murine tumors in vivo. *J Immunol* 1987; 138: 963–74.
15. Palladino MA, Shalby MR, Krammer SM, Ferraiolo BL, Baughman RA, Deleo AB, Crase D, Marafino B, Aggarwal BB, Figari IS, Liggitt D, Patton JS. Characterization of the antitumor activities of human tumor necrosis factor-α and the comparison with other cytokines: induction of tumor-specific immunity. *J Immunol* 1987; 138: 4023–32.
16. Okuyama M, Yamaguchi S, Yamaoka M, Nitobe J, Fujii S, Yoshimura T, Tomoike H. Nitric oxide enhances expression and shedding of tumor necrosis factor receptor I (p55) in endothelial cells. *Arterioscler Thromb Vasc Biol* 2000; 20: 1506–11.
17. Leek RD, Hunt NC, Landers RJ, Lewis CE, Royds JA, Harris AL. Macrophage infiltration is associated with VEGF and EGFR expression in breast cancer. *J Pathol* 2000; 190: 430–6.
18. De-Assis MC, Da-Costa AO, Barja-Fidalgo TC, Plotkowski MC. Human endothelial cells are activated by interferon-gamma plus tumour necrosis factor-alpha to kill intracellular *Pseudomonas aeruginosa*. *Immunology* 2000; 101: 271–8.
19. Nathan I, Dizdaroglu M, Bernstein L, Junker U, Lee C, Muegge K, Durum SK. Induction of oxidative DNA damage in u937 cells by TNF or anti-Fas stimulation. *Cytokine* 2000; 12: 881–7.
20. Ogata N, Yamamoto H, Kugiyama K, Yasue H, Miyamoto E. Involvement of protein kinase C in superoxide anion-induced activation of nuclear factor-kappa B in human endothelial cells. *Cardiovasc Res* 2000; 45: 513–21.
21. Mazanet R, Antman KH. Sarcomas of soft tissue and bone. *Cancer* 1991; 68: 463–73.
